# Cyto- and Genotoxicity of Selected Plant Extracts and Microbial Metabolites with Confirmed Activity Against Phytopathogens of Potato Seed (*Solanum tuberosum* L.)

**DOI:** 10.3390/molecules30030701

**Published:** 2025-02-05

**Authors:** Adriana Nowak, Aleksandra Steglińska, Beata Gutarowska, Dorota Kręgiel

**Affiliations:** Department of Environmental Biotechnology, Lodz University of Technology, Wólczańska 171/173, 90-530 Łódź, Poland; ale.steglinska@gmail.com (A.S.); beata.gutarowska@p.lodz.pl (B.G.); dorota.kregiel@p.lodz.pl (D.K.)

**Keywords:** cytotoxicity, genotoxicity, plant extracts, potato (*Solanum tuberosum* L.), insect cell line (Sf-9)

## Abstract

The aim of this study was to evaluate the cytotoxicity and genotoxicity of potential biocontrol agents for use against phytopathogens of potato seed (*Solanum tuberosum* L.). Plant extracts from *Allium sativum* L., *Syzygium aromaticum* L. Merr. & Perry, *Salvia officinalis* L., and *Curcuma longa* L., as well as metabolites of bacteria *Lactiplantibacillus plantarum* KB2 LAB 03 and yeast *Metschnikowia pulcherrima* TK1, were investigated. The chemical characteristics of the plant extracts and the metabolic profiles of the tested microorganisms were evaluated by GC-MS. An insect cell line from *Spodoptera frugiperda* (Sf-9) and human cervix adenocarcinoma cells (HeLa) were used to evaluate cytotoxicity in the 3-(4,5-dimethylthiazol-2-yl)-2,5-diphenyltetrazolium bromide (MTT) assay. The single-cell electrophoresis assay was used to estimate DNA damage. The cytotoxicity and genotoxicity of the microbial metabolites depended on their chemical profiles and pH. The plant extracts induced stronger DNA damage in the Sf-9 cell line than in HeLa cells. The garlic (*Allium sativum* L.) extract showed the highest cytotoxicity against Sf-9 insect cells (IC_50_ 41.6 mg/mL). The sage (*Salvia officinalis* L.) extract showed the highest cytotoxicity against HeLa cells (IC_50_ 49.6 mg/mL). This study is the first to investigate not only the potential of these novel biocontrol agents for plant disease control, but also their safety for humans and biodiversity within the context of sustainable agriculture.

## 1. Introduction

Phytopathogens pose a serious threat to crop productivity worldwide. Current anti-phytopathogen strategies include the development of disease-resistant crops and the application of chemical pesticides. These approaches have contributed to increased crop output and quality over the past few decades. However, the excessive use of toxic chemical compounds has resulted in environmental pollution and a drastic reduction in biodiversity [1]. Prolonged use of chemical treatments during plant cultivation has also resulted in resistance against fungal pathogens [2]. For these reasons, researchers are exploring the use of beneficial microorganisms as an eco-friendly strategy to control crop diseases. A wide range of bacterial and fungal genera show great potential as biocontrol agents for various plant diseases. Beneficial microbes are abundant sources of various natural compounds with the ability to control plant diseases at various levels [1]. These include primary and secondary metabolites: antibiotics, toxins, ribosomal or non-ribosomal peptides, polyketides, and volatile organic compounds.

Lactic acid bacteria (LAB) have the status of generally recognized as safe (GRAS), and are used as natural preservatives in food and feed to control fungal growth and subsequent mycotoxin production [3,4]. Previous studies by our team confirmed the ability of selected LAB to effectively inhibit the growth of fungal phytopathogens of seed potato (*Solanum tuberosum* L.). The best results were achieved with the strain *Lactiplantibacillus plantarum*, resulting in a 40–90% reduction in infestation by eight potato pathogens [5].

The *Metschnikowia pulcherrima* clade of yeast is considered an effective biocontrol agent against pathogenic moulds, mainly due to its ability to bind iron into a complex called pulcherrimin. The lack of free iron in the environment inhibits the growth of many microorganisms [6]. Pulcherrimin-producing yeasts exhibit strong antagonism against moulds belonging to the genera *Alternaria*, *Botrytis*, *Fusarium*, *Rhizopus*, and *Verticillium* [7]. Many studies have shown that the *M. pulcherrima* clade can effectively inhibit the growth of seed potato phytopathogens [8].

Plants are a valuable source of bioactive compounds, including terpenes, phenolic compounds, essential oils, and alkaloids. Numerous studies have investigated the effects of various plant extracts on plant pathogens. Due to the antimicrobial activity, biodegradability, and low toxicity of plant extracts, they can potentially be used in plant protection against pathogens instead of chemical pesticides [9]. In a recent study, garlic water extract with 5-hydroxymethylfurfural (5-HMF) as the main component proved to be particularly effective as a potential biopesticide against potato phytopathogens [10]. In another study, all the studied microorganisms and plant extracts not only inhibited the growth of the tested phytopathogens, but also had positive effects on the physiological parameters of potatoes and reduced the production of mycotoxins by *Fusarium*, *Alternaria*, and *Phoma* [11].

Microorganisms and natural substances of plant origin are considered to have less impact on ecosystems and human health than conventional chemicals for plant protection. However, to ensure the sustainability of biocontrol solutions, it is necessary to understand their possible negative repercussions. The use of living organisms, capable of growth and metabolite production, as biocontrol agents presents specific challenges compared to conventional solutions, because these microorganisms can survive, multiply, move, and effectively colonize other environments. To date, only a limited number of studies have addressed the presence of biocontrol agents in the environment, their environmental fate, and their effects on biodiversity. However their results suggest that while most have low ecotoxicity, others have toxicity equivalent to or greater than conventional solutions. Overall, knowledge of the unintended effects of biocontrol agents in the environment is very incomplete [12].

The purpose of this study was to investigate the potential ecotoxicological impact of selected biocontrol agents on other useful living organisms in the environment and on biodiversity. The main aim was to evaluate the cytotoxicity and genotoxicity of selected plant extracts: garlic (*Allium sativum* L.), clove (*Syzygium aromaticum* L. Merr. & Perry), sage (*Salvia officinalis* L.), and turmeric (*Curcuma longa* L.). We also evaluated the toxicity of metabolites of *L. plantarum* and *M. pulcherrima*, as potential biocontrol agents for potato seeds (*Solanum tuberosum* L.). Insect cell lines were used, among other methods, as an effective model to investigate the toxicological mechanisms of the biocontrol agents [13]. This research is part of a new trend for developing innovative strategies using diverse biological agents, such as plant extracts and native microorganisms, as well as examining their impact on the environment.

## 2. Results and Discussion

### 2.1. Chemical Composition of Aqueous Plant Extracts

In our previous research, we determined the compositions of garlic (*Allium sativum* L.) and clove (*Syzygium aromaticum* L. Merr. & Perry) aqueous extracts [10]. The main components of the clove extract were eugenol and eugenol acetate, amounting to 82.39% and 4.56% of the total composition, respectively. The garlic extract was dominated by 5-HMF (33.24%), followed by furan-2-carboxaldehyde (7.09%), acetaldehyde (5.17%), and 3-methylbutanal (4.27%) [10]. The other aqueous extracts used in the present study were sage (*Salvia officinalis* L.) and turmeric (*Curcuma longa* L.). The volatile components of the plant extracts were analysed and identified by gas chromatography–mass spectrometry (GC-MS) combined with Kovats retention index. This is a commonly used method for the characterization of volatile compounds in various plant extracts [14]. The chemical compositions of the tested extracts are presented in Table 1 and Table 2. The chromatograms show great variation in the chemical profiles of the plant extracts tested. The most important chemical compounds are listed int the tables, but the synergistic effects of other compounds should not be excluded.

**Figure 1:**
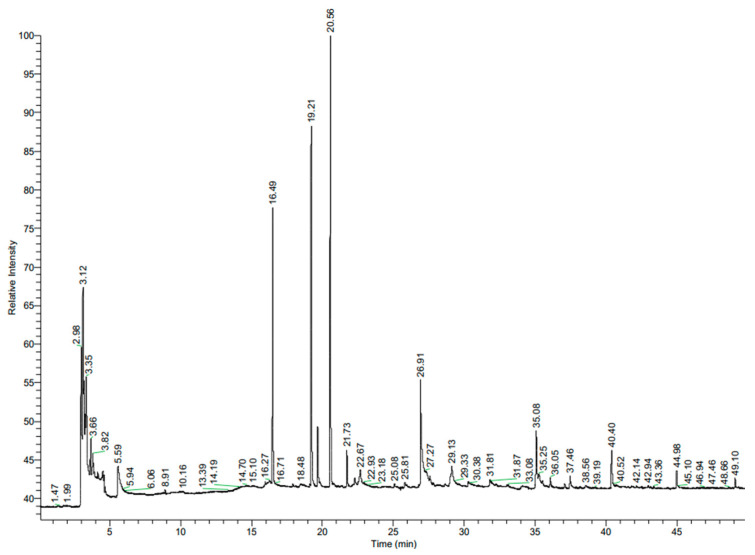
Chromatogram of sage (*Salvia officinalis* L.) aqueous extract.

*Salvia* spp. belong to the family of Lamiaceae and are associated with potent antimicrobial activity, primarily due to their chemical constituents [15,16]. According the Drugs and Lactation Database LactMed^®^ [17], sage leaves contain mainly tannins (salviatannin), essential oils (including α-thujone, *β*-thujone, 1,8 cineole, and camphor), flavones, phenolic acids, phenylpropanoid glycosides, triterpenoids, and diterpenes. The tested sage extract was dominated by camphor (31%), α-thujone (24%), and eucalyptol (18%) (Table 1, Figure 1).

The antimicrobial activity of these compounds is well known [18,19]. Sage essential oil is active against the mycelial growth of the strawberry pathogens *Colletotrichum acutatum* [20], *Alternaria alternata*, *Botrytis cinerea*, and *Fusarium oxysporum* [16]. It is worth noting the presence in the profile of 2-metoksy-4-vinylphenol (11%). This compound often causes severe skin burns and eye damage [21].

**Figure 2:**
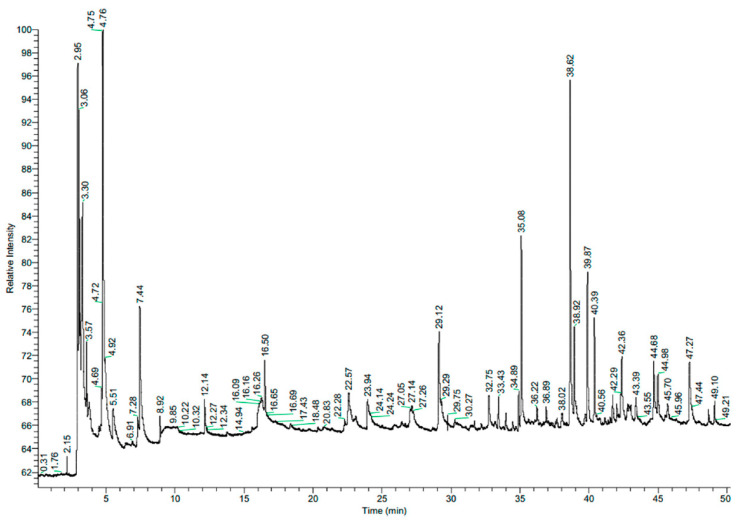
Chromatogram of turmeric (*Curcuma longa* L.) aqueous extract.

*Curcuma longa* (turmeric) belongs to the family Zingiberaceae. Turmeric essential oil acts as a natural food preservative and alternative to chemical fungicides. It has been shown to have a significant antifungal effect against *Penicillium expansum*, *Fusarium solani*, *Rhizopus stolonifera*, and *Alternaria alternata* [22]. In our study, the turmeric extract was composed mainly of 1-hydroxy-2-propanone (acetol) (21%), ar-turmerone (19%), and ethanol (14%) (Table 2, Figure 2).

These compounds have known potential antimicrobial activity [23,24]. However, it is worth noting the significant share (17%) of unidentified compounds in the profile. The effects of these compounds on living organisms are unknown.

### 2.2. Cytotoxicity of Plant Aqueous Extracts

Sf-9 (*Spodoptera frugiperda*) and HeLa cells were exposed to the extracts for 24 h. Non-cytotoxic concentrations (IC_0_) and IC_50_ were determined for all extracts and both cell lines (except for the garlic extract, for which only the IC_20_ value was determined due to its weak cytotoxicity towards HeLa cells). Against the Sf-9 insect cell line, the extracts with the highest cytotoxicity were garlic (IC_50_ 41.6 mg/mL), followed by turmeric and sage, and the weakest cytotoxicity was shown by clove (Table 3). Against the model HeLa cell line, sage extract showed the strongest cytotoxicity (IC_50_ 49.6 mg/mL), followed by clove, turmeric, and finally garlic. Overall, the garlic and turmeric extracts showed stronger cytotoxicity against Sf-9 insect cells than against HeLa cells. λ-Cyhalothrin always showed the highest cytotoxicity, which was more than 200 times stronger than the cytotoxicity of the garlic extract against Sf-9 cells.

*S. frugiperda* insects are common pests of plants belonging to the Solanaceae Juss. family. Sf-9 cells are the most established insect cells. They are used in agricultural research and toxicity studies. They are particularly valuable for assessing the ecotoxicity of environmental pollutants, studying the control of plant pests and crop protection strategies (especially in mass production systems), evaluating the cytotoxicity of insecticides, and supporting the development of new biopesticides against crop pests [25]. In vitro studies using Sf-9 cells allow for rapid toxicity screening of biopreparations and their components.

The cytotoxicity and mechanism of cytotoxic action of garlic extracts have been investigated for many human cell lines, including tongue squamous carcinoma (SCC-15), normal skin fibroblasts (BJ), epithelial papilloma (KB), leukemic (U-937, Jurkat, E6-1, K-562, TIB-152), cervix adenocarcinoma (HeLa), colorectal cancer (DLD-1), lung squamous carcinoma (SK-MES-1), colon adenocarcinoma (Caco-2), hepatic carcinoma (Hep-G2), prostate cancer (PC-3), breast cancer(MCF-7), and normal human keratinocytes (HaCaT) [26,27,28,29,30,31,32]. The main mechanisms of the cytotoxic action of garlic extracts are the induction of reactive oxygen species (ROS) generation, inhibition of proliferation, and cell death by necrosis or apoptosis [27,28,29,32]. In our study, we used the 3-(4,5-dimethylthiazol-2-yl)-2,5-diphenyltetrazolium bromide (MTT) assay to evaluate cytotoxicity. The MTT assay is widely used to measure cell metabolic activity, which is an indirect indicator of cell viability. The high cytotoxicity of the garlic extract in our study may be due to the content of acetaldehyde. Although it did not account for a large proportion of the extract’s composition (about 5.2%), acetaldehyde can exhibit strong mitophagy-related cytotoxicity, which involves the removal of damaged mitochondria through lysosomal degradation. The results of the MTT assay may also reflect impaired and reduced mitochondrial function after exposure to this compound [33]. This may be linked to a decrease in mitochondrial membrane potential (MMP), ROS production, and the ATP level in the cell [33,34]. Tigu et al. [32] obtained IC_50_ values ranging from about 8.8 to 63 mg/mL, depending on the cell line. In our study, the IC_50_ value for the Sf-9 cells was within this range, at 41.6 mg/mL. As in the study by Tigu et al., normal cells were much more sensitive to garlic extract than cancerous cells.

The cytotoxic potential of clove extracts has likewise been tested on a number of human cell lines, including colon adenocarcinoma (HCT 116, HT-29), breast adenocarcinoma (MCF-7, AU 565), HeLa, Hep-G2, and lung alveolar carcinoma (A-549) [35,36,37,38,39,40,41]. The main mechanism of cytotoxic action of clove extract is the induction of apoptosis. The main component responsible for cytotoxic effect is eugenol [42]. In our study, eugenol and eugenol acetate accounted for more than 86% of all components in the clove extracts. Various extracts from different parts of clove (e.g., leaves, stems, fruits, buds, rhizomes) display cytotoxic effects on cell lines by inhibiting proliferation, inducing ROS, and causing cell cycle arrest [43].

The cytotoxicity of curcumin may be related to its uptake into the cell. As a lipophilic molecule, curcumin interacts with the cell membrane and is then transported into the cell [44]. The more curcumin enters the cell, the greater its cytotoxicity. This mechanism has been demonstrated for cell lines including mouse T lymphoblast EL4, human MCF-7, and murine fibroblasts NIH3T3 [44]. Ar-turmerone accounted for almost 19% of the components in the studied turmeric extract. This compound has been shown to contribute to the uptake of curcumin into the cell in vitro [45]. Many other studies have reported that curcumin inhibits the growth of various cells, including mouse skin melanoma—B16-F1, human colon adenocarcinoma—COLO 205, HCT 116, and Caco-2, human hepatic stellate—LX-2, Hep-G2, and HeLa, in assays based on tetrazolium salts (MTT, MTS, WST-1) [46,47,48,49]. In HeLa cells treated with curcumin, apoptotic changes and cell cycle arrest (in G0/G1 and S phases) were observed in proportion to its concentration [49]. Ar-turmerone may be responsible for this cytotoxic effect and contribute to the induction of apoptosis in cells [50]. Curcumin-based secondary metabolites have well-documented potent cytotoxic activities [51]. Curcumin-based linear diarylheptanoids 35a and 35b have been shown to display antiproliferative activity on human cancer cells (MCF-7 and Hep-G2). Furthermore, 35b increases GADD45B (growth arrest and DNA-damage-inducible, beta) expression, leading to inhibition of MCF-7 proliferation and death. The same study confirmed the importance of the conjugated group for antiproliferative activity [51]. Some linear diarylheptanoids, due to their chemical structure (at least two substituents on aryl rings), demonstrate higher cytotoxic and anticancer properties against cancer cell lines than curcumin [52]. Sage extract may be cytotoxic to Hep-G2 cells, causing morphological changes in cells and inhibiting proliferation, while decreasing the intracellular ATP level, lactate dehydrogenase (LDH) leakage, and apoptosis [53]. Sage extract can also induce apoptosis in human lymphoma and leukaemia cells [54] and show cytotoxicity against other human-derived cell lines, including HeLa and MCF-7 [55]. One of the main components of the tested sage extract was eucalyptol, which can display cytotoxicity in cells by inducing apoptosis [56]. Other components included camphor and α-thujone. All these compounds (camphor, eucalyptol, α-thujone) can be cytotoxic to colon carcinoma cells (MRC-5, HT-29, and HCT 116) [57].

To the authors’ best knowledge, the studied aqueous extracts have not previously been considered as bioinsecticides against Sf-9 cells. Lopes et al. [58] investigated seven different plant extracts (in water, ethanol, or dichloromethane as solvents) against Sf-9 cells, observing that the less polar samples were more cytotoxic. Dichloromethane extract caused a loss of cell viability that exceeded the effect of the commercial insecticide chlorpyrifos. In our study, λ-cyhalothrin always showed more cytotoxicity than the tested extracts. λ-Cyhalothrin belongs to the class of Type II pyrethroid insecticides. This compound has insecticidal properties and is used to control a wide range of insect pests and diseases. It is one of the most powerful pyrethroid insecticides in the world. Due to its acute toxicity, the insecticide is extremely toxic in mice and induces hepatic and renal toxicity in rats. In cells in vitro it induces micronuclei, nucleoplasmic bridges, nuclear buds, necrosis, and apoptosis, which is positively related with carcinogenicity [59]. The main mechanisms of cytotoxicity of λ-cyhalothrin are hepatotoxicity, neurotoxicity, nephrotoxicity, and reproductive toxicity, as well as induction of oxidative stress leading to mitochondrial, lipid, DNA, and protein damage in cells [60]. The overall mechanism of toxic action of λ-cyhalothrin is due to its chemical structure. However, this mechanism is not fully understood and is the subject of systematic research [60].

### 2.3. Cytotoxicity of Microbial Metabolites

Sf-9 and HeLa cells were exposed to the microbial metabolites for 48 h. *L. plantarum* KB2 LAB 03 metabolites were more cytotoxic to Sf-9 cells at physiological pH than at neutral pH (Table 4). *L. plantarum* KB2 LAB 03 metabolites showed greater cytotoxicity after culturing in MRS than after culturing in AM at physiological pH. The cytotoxicity of *L. plantarum* KB2 LAB 03 fermentation by-products against the HeLa cell line showed less variation. However, the metabolites cultured in MRS also showed greater cytotoxicity than those cultured in AM. Similarly, greater cytotoxicity was observed for yeast metabolites towards Sf-9 cells at physiological pH. The highest cytotoxicity was observed for metabolites cultured on sYPG medium and the lowest after culturing in sYP. In the case of the metabolites cultured on sYPG, no cytotoxicity was observed at pH 6.2, but an enhanced pro-proliferative effect was noted. Yeast cultured in sYP at both pH options had the same effect on HeLa cells (Table 4). The yeast metabolites cultured in sYPG also showed the strongest cytotoxicity towards HeLa cells.

In our previous studies, we identified the main metabolites of *L. plantarum* KB2 LAB 03 after cultivation in MRS medium. The metabolites were mostly lactic, acetic, and isobutyric acids. The main fermentation by-products of the strain cultured in AM (acid whey-based) medium were lactic, acetic, and propionic acids, as well as ethanol [5]. We also studied the main metabolites of *M. pulcherrima* TK1 after cultivation in YPD medium. The main metabolites were ethanol and glycerol, as well as lactic, succinic, and acetic acids. After fermentation in sYP (acid whey-based) medium, the by-products were lactic acid, ethanol, glycerol, acetic, and succinic acids [8]. Organic acids such as propionic acid can also suppress the viability of HeLa cells, by inducing ROS generation and dysfunction of the mitochondrial membrane, leading to autophagy [61]. Elsewhere, we showed that pure lactic, acetic, and propionic acids inhibit the growth of Caco-2 cells [62]. We screened the cytotoxicity of metabolites of 39 LAB strains against Caco-2 cells (at neutralised pH). The metabolite concentrations ranged from 10 to 200 mg/mL. Cytotoxicity depended on the concentration of metabolites: the higher their concentration, the higher the cytotoxicity.

In the current research, we found LAB metabolite concentrations ranging from 0.001 to 200 mg/mL. Their cytotoxic effect may be related to the acidic pH of some LAB metabolites. In the case of Sf-9 cells, cytotoxicity increased at lower sample pH. Such a relationship was not observed for HeLa cells cultivated with LAB fermentation by-products and not at all for yeast metabolites. This may indicate that a different mechanism is responsible for their cytotoxicity. It has been suggested that the protein nature of secreted metabolites may be responsible for their cytotoxicity [63]. According to the literature, several cell components (cytoplasmic fractions, fermentation by-products including organic acids, cell wall components, exopolysaccharides (EPS), peptidoglycan, conjugated linoleic acids, and S-layer proteins) can have strong cytotoxic effects on various cell lines [64,65,66]. Bacteriocins such as plantaricin A produced by *L. plantarum* and enzymes such as arginine deiminase produced by some strains of LAB can also inhibit the growth of some cells in vitro [66]. Cell wall components of yeast, β-glucan and chitin, can have the same effect [67,68]. Generally, the cytotoxicity of microbial fermentation by-products depends on the strain being tested, the cell line, and the culture medium. The main mechanisms of their cytotoxic activity are cell cycle arrest, apoptosis, necrosis, increased ROS production, and decreased intracellular ATP and MMP [60,62,69].

### 2.4. Genotoxicity of Plant Aqueous Extracts

After screening for cytotoxicity, extracts with concentrations close to or below the IC_50_ values were selected for further testing for genotoxicity in the comet assay (Table 5 and Table 6). Due to their different cytotoxic activity against the two cell lines, different concentrations were used. Concentrations higher than the IC_50_ values induced severe, unmeasurable DNA damage (apoptotic cells), so were not tested. Due to the wide variety of concentrations tested in the cytotoxicity assays, they could not be standardized in genotoxicity tests.

The rate of DNA damage in the negative controls was 2.35% ± 5.74% (for HeLa cells) and 4.54% ± 0.48% (for Sf-9 cells). At the tested concentration of 1.3 mg/mL, λ-cyhalothrin was 4-fold more cytotoxic against Sf-9 cells than HeLa cells. It was noticed that λ-cyhalothrin always displayed the strongest genotoxicity, which was also greater against insect Sf-9 cells than human HeLa cells. The genotoxicity of λ-cyhalothrin against Sf-9 cells was 42.32% ± 3.59% at a concentration of 0.63 mg/mL and 42.62% ± 3.31% at a concentration of 1.3 mg/mL. Against HeLa cells, its genotoxicity was 10.46% ± 2.56% at 1.3 mg/mL and 35.20% ± 4.00% for concentrations of 1.3 and 2.5 mg/mL, respectively.

**Figure 3:**
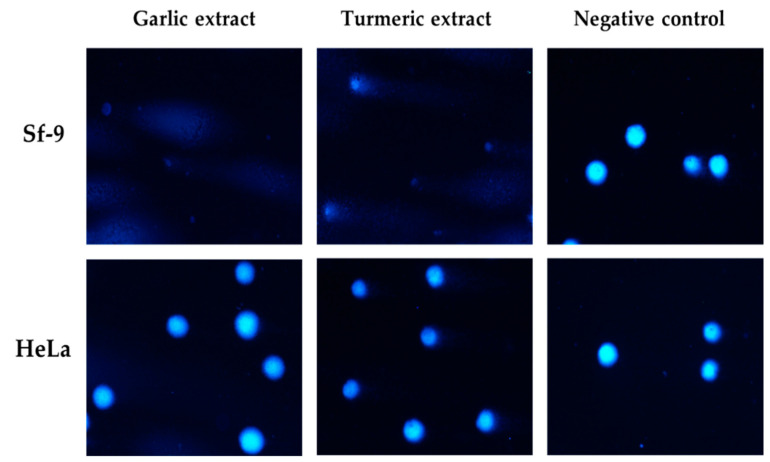
Representative photographs of comet cells after exposure to garlic and turmeric aqueous extracts (62.5 mg/mL), stained with DAPI, fluorescence microscopy (Nikon, Tokyo, Japan), 20× objective.

Sf-9 cells were more sensitive to garlic and turmeric extracts than HeLa cells at similar concentrations, and genotoxicity was not proportional to concentration. Garlic extract showed the highest genotoxicity against Sf-9 cells (close to 50%), and clove extract showed the highest genotoxicity against HeLa cells (up to 44.55%) at a concentration of 50 mg/mL (Figure 3).

The variability of the raw material and extracted components is a significant factor that limits studies on the toxicity of extracts from different plants. Aqueous and alcoholic garlic extracts have been shown to induce genotoxicity in peripheral blood lymphocytes, as evaluated in the comet assay, and cause chromosome aberrations in human leucocytes [70,71]. The main component of our garlic extract was 5-HMF, which can demonstrate genotoxicity at higher concentrations [72]. 5-HMF can induce DNA damage in Hep-G2 cells, suggesting a weak genotoxic effect, probably due to its rapid cell repair capacity [73]. This weak genotoxic effect may explain why in our study HeLa cells were practically resistant to the activity of garlic extract. According to the literature, eugenol—the main component of clove extract—can induce moderate to severe genotoxic effects in hamster lung fibroblasts V79, in a dose-dependent way [74]. In our study, eugenol may have been responsible for the medium genotoxicity of clove extract against the tested cells. Beltzig et al. [75] investigated the genotoxicity of ethanolic solvent and micellar curcumin against several primary and cancerous cell lines. They showed that curcumin had similar genotoxic effects against the cancerous cell lines, but also observed high efficiency of DNA repair after its removal. Crude extract of *Curcuma longa* L. demonstrates genotoxic activity against genomic DNA in the human tumour cell lines: prostate DU-145 and colon HT-29 [76]. Curcumin at high concentrations displayed a significant increase in MN frequency in the micronucleus (MN) test [77]. The main components of sage extract, camphor, eucalyptol, and α-thujone induced evident DNA breaks in human colon HT-29 and HCT 116 cells, foetal lung fibroblasts MRC-5, and Vero (kidney monkey) cells [57]. These compounds may be responsible for the genotoxicity of sage extract observed in our study.

### 2.5. Genotoxicity of Microbial Metabolites

**Figure 4:**
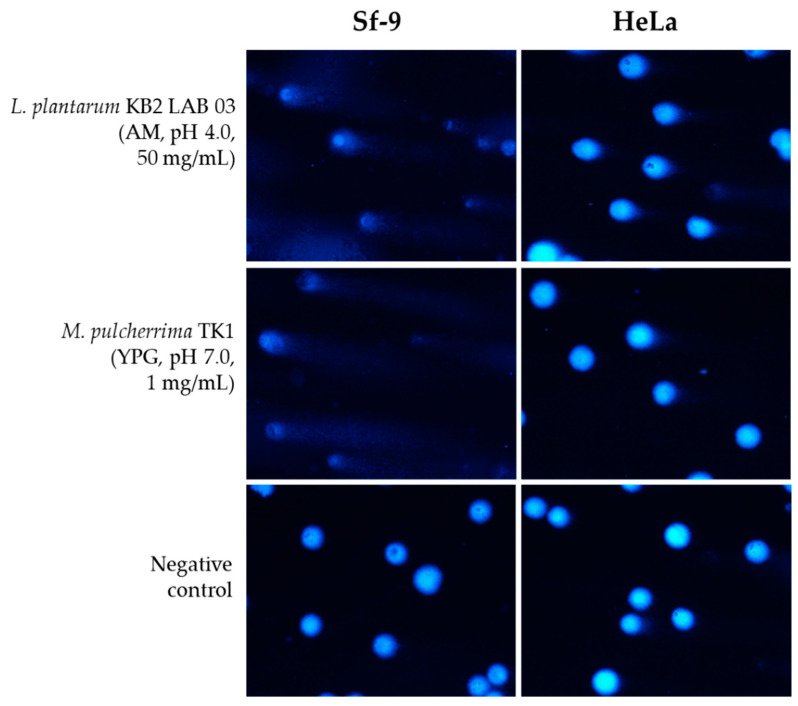
Representative photographs of comet cells after exposure to *L. plantarum* KB2 LAB 03 and *M. pulcherrima* TK1 exemplary metabolites, stained with DAPI, fluorescence microscopy (Nikon, Tokyo, Japan), 20× objective.

After screening for cytotoxicity, extract concentrations close to or below the IC_50_ value were selected for further testing (Table 7, Table 8, Table 9 and Table 10, Figure 4). Due to their different cytotoxic activity against the two cell lines, the tested concentrations were also different. Concentrations higher than the IC_50_ values induced very strong unmeasurable DNA damage (including apoptotic cells), so they were not used. In general, the genotoxicity of the bacterial metabolites was not dependent on the pH or concentration (Table 7). The genotoxicity of the metabolites against Sf-9 cells was not dependent on their concentration and remained at similar levels. Metabolites of *L. plantarum* in AM at physiological pH showed the highest genotoxicity (54.52 ± 4.45%) at a concentration of 50 mg/mL compared to the other bacterial samples (*p* ≤ 0.05). Yeast metabolites showed generally higher genotoxicity than LAB metabolites. Independently of pH, yeast metabolites in sYPG showed the strongest genotoxicity (Table 8), which was correlated with cytotoxicity. On YPG, greater genotoxicity was observed at physiological (acidic) pH.

The bacterial and yeast metabolites showed no or weak genotoxicity against HeLa cells, compared to the Sf-9 line (Table 9 and Table 10). Genotoxicity was weakly or uncorrelated with metabolite concentration. Metabolites of *L. plantarum* KB2 LAB 03 in MRS showed the highest but weak genotoxicity regardless of pH, and in AM these metabolites showed the lowest genotoxicity. In the case of HeLa cells, yeast metabolites showed greater genotoxicity than LAB metabolites. Yeast metabolites in sYPG medium showed the weakest genotoxicity at the tested concentrations.

To the best knowledge of the authors, the genotoxicity of by-products of fermentation by LAB and yeast on cell lines has not previously been studied. Since one of the mechanisms for the toxic effects of the tested microbial fermentation by-products may be increased intracellular ROS production, they can induce oxidative damage to DNA, leading to apoptosis [65,78]. Ethanol can also be genotoxic, as was shown in a comet assay using peripheral blood lymphocytes as well as human primary gastric and colon mucosa [79].

## 3. Materials and Methods

### 3.1. Biopreparations Design from Lactiplantibacillus Plantarum KB2 LAB 03 and Metschnikowia pulcherrima TK1

Analyses were conducted according to the method described elsewhere [11]. Briefly, *Lactiplantibacillus plantarum* KB2 LAB 03 and *Metschnikowia pulcherrima* TK1 strains were used to obtain bacterial biopreparations against potato phytopathogens [5,10]. These strains were selected based on our previous studies, as they produced the most bioactive metabolites that inhibited the growth of potato phytopathogens. The strains were stored in Cryobanks™ at −20 °C. Before the experiments, they were activated, threefold passaged, and cultured. *L. plantarum* KB2 LAB 03 were cultured in deMan–Rogosa–Sharp (MRS, Merck Life Science, Poznań, Poland) broth for 24 h at 30 °C. *M. pulcherrima* TK1 were cultured in YPG (Yeast Extract, Peptone, Glucose; BTL, Warsaw, Poland) broth for 72 h at 25 °C on a shaker (Unimax 1010, Schwabach, Germany) at 160 rpm.

*L. plantarum* KB2 LAB 03 was also activated and cultured on an acid whey-based medium (abbreviated as AM) supplemented with 1.4% peptone K, 0.8% yeast extract (BTL, Poland), 0.2% dipotassium phosphate (Chempur, Poland), 0.2% ammonium citrate (Chempur, Poland), 0.02% magnesium sulphate heptahydrate (Chempur, Poland), 0.5% sodium acetate (Chempur, Poland), and 0.005% magnesium sulphate tetrahydrate (Chempur, Poland). The bacteria were cultured for 48 h at 30 °C.

*M. pulcherrima* TK1 was also activated and cultured on an acid whey-based medium supplemented with 1% glucose, 0.25% peptone, and 0.13% yeast extract (abbreviated as sYPG) or 0.25% peptone and 0.25% yeast extract (abbreviated as sYP) for 72 h at 25 °C on a shaker at 160 rpm.

After incubation, the samples were centrifuged (10,733× *g*, 15 min) and decanted. Two supernatant options were used for cyto- and genotoxicity testing—i.e., at physiological pH and after neutralization. The pH was adjusted to 7.0 ± 0.1 (with 0.1 M NaOH and HCl). Finally, the supernatants were filtered with sterile 0.22 μm pore size syringe filters and frozen at −20 °C until analysis.

As a result, several variants of supernatants (post-fermentation fluids) were obtained after microbial cultivation of:

- *L. plantarum* KB2 LAB 03 in MRS at pH 4.0 and 7.0;
- *L. plantarum* KB2 LAB 03 in AM at pH 4.0 and 7.0;
- *M. pulcherrima* TK1 in YPG at pH 5.9 and 7.0;
- *M. pulcherrima* TK1 in sYP at pH 6.2 and 7.0;
- *M. pulcherrima* TK1 in sYPG at pH 5.4 and 7.0.

By-products of *L. plantarum* KB2 LAB 03 and *M. pulcherrima* TK1 fermentation on the abovementioned media were determined in our previous studies using high-performance liquid chromatography (HPLC) [5,8].

### 3.2. Design of Biopreparations from Plant Extracts

#### 3.2.1. Plant Material and Extraction Conditions

Plant material was purchased from Dary Natury Sp. z o. o., Grodzisk, Poland, and Herbal Plant KAWON–HURT Nowak sp.j., Gostyń, Poland. The plant material consisted of sage (*Salvia officinalis* L.) leaves and stems, turmeric (*Curcuma longa* L.) roots, garlic (*Allium sativum* L.) bulbs, and clove (*Syzygium aromaticum* L. Merr. & Perry) flower buds. The aqueous plant extracts were prepared as described elsewhere [11]. First, 50 g of finely ground material was poured into 500 mL of water at 100 °C and left covered without stirring for 1 h. The plant extracts were then sonicated (40 kHz, 25 °C, 30 min) and filtered under reduced pressure.

#### 3.2.2. Gas Chromatography Mass Spectrometry (GC-MS) Analysis of Chemical Composition

The volatile components extracted from the plant extracts were analysed and identified by gas chromatography–mass spectrometry(GC–MS) combined with Kovats retention index. All data were compared to Wiley, Adams, and Nist libraries. Gas chromatography–mass spectrometry (GC–MS) analyses were conducted as described elsewhere [10], using a Thermo Ultra GC Trace with a flame ionization detector, a Thermo DSQ II mass spectrometer (split flow), and Rxi^®^—1 ms (60 m × 0.25 mm × 0.25 µm film thickness) column from RESTEK. The detector temperature (FID) was 300 °C, the injector temperature was 280 °C. The temperature of the transfer line was 280 °C. The temperature of the ion source was 220 °C. The injection quantity was 1 µL with a 15 mL/min split for aqueous extract samples, and 0.5 µL with a 100 mL/min split for CO_2_ extract samples. Based on electron spectra from the NIST 2011 library and the Kovats index, the volatile compounds were identified.

### 3.3. Cell Cultures

The insect cell line from pupa ovarian tissues *Spodoptera frugiperda* (Sf-9, fall armyworm) was cultured as a monolayer in ready-to-use Sf-900™ III Serum-Free Medium (Thermo Fisher Scientific, Waltham, MA, USA) at 27 °C, in a non-humidified ambient air-regulated incubator (Binder BD 56, GmbH, Tuttlingen, Germany) for 10–14 days until it reached 80% confluence. HeLa (human cervix adenocarcinoma) cells were cultured in high-glucose DMEM (Merck Life Science, Poland), with the addition of 10% fetal bovine serum (FBS), 4 mM GlutaMAX^TM^ (Thermo Fisher Scientific, Waltham, MA, USA), 25 mM 4-(2-hydroxyethyl)-1-piperazineethanesulphonic acid (HEPES) (Merck Life Science, Poland), and 100 µg/mL streptomycin/100 IU/mL penicillin (Merck Life Science, Poland). The Sf-9 cells were purchased from ThermoFisher Scientific, Carlsbad, CA, USA (Lot No. 2177129), while the Caco-2 cells were purchased from Cell Line Services GmbH Eppelheim, Germany (Lot No. 300137-1514SF). Cells were cultured as a monolayer at 37 °C, with 5% CO_2_, in a humidified incubator (Galaxy 48S, New Brunswick, United Kingdom) for 3–5 days, until they reached 80% confluence. Every 2–3 days, the cells were washed with phosphate buffer saline (PBS, Merck Life Science, Poland) (pH 7.2) without calcium and magnesium, and the medium was renewed. The confluent Sf-9 cells were detached from the substrate by gentle pipetting, centrifuged (200× *g*, 5 min), and decanted. The pellet was resuspended in a fresh culture medium. The confluent HeLa cells were detached with TrypLE^TM^ Express (Thermo Fisher Scientific, Waltham, MA, USA) (37 °C, 8–10 min), centrifuged (307× *g*, 5 min), and decanted. The pellet was resuspended in a fresh culture medium. After the detachment procedure, a cell count was performed using a hemocytometer and cell viability was determined by trypan blue exclusion. The cells were then ready to use. The viability of the cells used in the experiments was at least 80% (Sf-9) and 90% (HeLa).

### 3.4. 3-(4,5-Dimethylthiazol-2-yl)-2,5-diphenyltetrazolium Bromide (MTT) Assay

The cytotoxicity of the samples was assessed using the MTT assay. A total of 5000 (HeLa) or 20,000 (Sf-9) cells/well were placed in 96-well plates and incubated for 24 h at 37 °C in 5% CO_2_ (HeLa) or at 27 °C in non-humidified incubator (Sf-9). The next day, the medium was removed and dilutions of the biopreparations and plant extracts were added on a cell monolayer. The final concentrations of the analysed samples were as follows (mg/mL): 0.39–250 (all plant extracts, Sf-9 cells; garlic and curcuma extracts, HeLa cells); 0.39–100 (sage and clove extracts, HeLa cells); 0.001–200 (metabolites of microorganisms for both cell lines). Each concentration was tested in four replicates. The cells were exposed to the extracts for 24 h and to the metabolites for 48 h. Efforts were made to select the exposure time and concentration in such a way as to determine the IC_0_ (non-toxic concentration) and IC_50_ values (the concentration of the test compound that reduces cell viability by 50% compared to the negative control). In the case of low cytotoxicity, the IC_20_ value was calculated accordingly. Several experiments were performed for the extracts in order to refine the method and determine the IC_50_ values. The experiment was repeated for comparison using the insecticide λ-cyhalothrin (Merck Life Science, Poland), which is used to control insect pathogens attacking potatoes at concentrations of 0.16–5.0 (Sf-9 cells) and 0.02–5.0 (HeLa cells). Negative controls were non-exposed cells.

After the exposure time, test samples were gently aspirated and MTT (0.5 mg/mL in PBS) was added to each well and incubated for further 3 h. The MTT was then aspirated and dimethyl sulfoxide (DMSO) (Merck Life Science, Poland) was added to dissolve the formazan crystals. Absorbance was measured at 550 nm (using a 620 nm reference filter) in a microplate reader (TriStar^2^ LB 942, Berthold Technologies GmbH & Co. KG, Bad Wildbad, Germany). Negative control absorbance represented 100% cell viability. Cell viability (%) was calculated as (sample OD/control OD) × 100%. Cytotoxicity (%) was calculated as 100-cell viability. The results are presented as the mean of four individual readings ± the standard deviation (±SD). The IC_0_, IC_20_, and IC_50_ values were estimated from the resulting curves.

### 3.5. Single-Cell Electrophoresis Assay (Comet Assay)

Eppendorf tubes were loaded with the appropriate cell culture medium (Sf-9 or HeLa), 1 × 10^5^ cells/sample and the test sample (plant extract or metabolites), so that the final volume was 1 mL. The final volume of the test sample was 20% (*v*/*v*). The concentrations of the test samples were selected on the basis of cytotoxicity tests. Samples with cytotoxicity ≤IC_50_ were selected. The final test concentrations were 1.25–125 mg/mL (Sf-9) and 1.56–250 mg/mL (HeLa) for the extracts and 0.01–100 (Sf-9) and 0.1–200 (HeLa) for metabolites. Cells suspended in the medium without the addition of compounds were used as a negative control. The samples were incubated for 60 min at 27 °C (Sf-9) and 37 °C (HeLa), then centrifuged (15 min, 4 °C, 182× *g*) and decanted. Low Melting Point (LMP) agarose (Merck Life Science, Poland) was added at 37 °C. The suspension was spotted on warm Normal Melting Point (NMP) agarose double-layered slides and covered with coverslips (hot plate ZF6 Premiere Slide Warmer). Next, the samples were placed on a Chilling Plate for Comet Assay Slides (Cleaver Scientific, Rugby, UK) and allowed to solidify. Alkaline lysis was performed with buffer (2.5 M NaCl, 1% Triton X-100, 100 mM EDTA, 10 mM Tris, pH 10), and the reaction mixture was incubated (60 min, 4 °C). The lysis buffer was decanted, and the slides were flooded with the unwinding buffer (300 mM NaOH, 1 mM EDTA) (20 min, 4 °C). The slides were then placed in an electrophoresis apparatus (CSL-COM20, Cleaver Scientific). Electrophoresis was performed in electrophoretic buffer (300 mM NaOH, 1 mM EDTA, pH > 13) for 20 min at a voltage of 21 V and a current of 29 mA. The slides were neutralized, allowed to dry, and then stained for 60 min at 4 °C with 4′,6-diamidino-2-phenylindole (DAPI) (1 µg/mL). Comet analysis was performed under a fluorescence microscope (Nikon) at a magnification of 200× equipped with a camera (Nikon Digital Sight DS-U3) and with Lucia Comet v.7.0 software (Laboratory Imaging, Prague, Czech Republic). In each trial, 50 randomly selected comets were analysed based on the parameter determining the percentage of DNA in the comet’s “tail”. The results are presented as the mean ± S.E.M.

### 3.6. Statistical Analysis

Statistical analysis was conducted using one-way ANOVA followed by Tukey’s multiple-comparisons post hoc test, performed using OriginPro 6.1 (Northampton, MA, USA) software at a significance level of *p* ≤ 0.05. Differences between samples with normal distributions were evaluated using a Student’s *t*-test.

## 4. Conclusions

The agricultural sector faces significant challenges due to the rising resistance to chemical pesticides and emerging environmental threats, which also have implications for public health. In this context, one of the most critical strategies in sustainable agriculture is the use of biocontrol microorganisms and natural plant extracts to suppress phytopathogens. Equally important is understanding the dual role of these biocontrol agents—not only in managing plant diseases but also in ensuring safety for humans and contributing to the preservation of biodiversity. In this study, we evaluated the cytotoxicity and genotoxicity of potential biocontrol agents for use against phytopathogens of potato seed (*Solanum tuberosum* L.). The tested plant extracts with potential biocontrol functions showed cytotoxicity and genotoxicity against both tested cell lines: the insect cell line Sf-9 and the human cell line HeLa. The most cytotoxic extract towards the Sf-9 line was garlic extract. The least cytotoxic was clove extract. Sage extract showed the highest cytotoxicity potential towards the HeLa cell line. Garlic extract showed the lowest cytotoxicity potential towards this cell line. Garlic and turmeric extracts showed stronger cytotoxicity towards Sf-9 insect cells than towards HeLa cells. Garlic extract showed the highest genotoxicity towards Sf-9 cells, and clove extract showed the highest genotoxicity towards HeLa cells. Both extracts and microbial metabolites showed stronger genotoxicity against Sf-9 cells (normal insect cells) than HeLa lines (model human cancer cells). The metabolic profiles of *M. pulcherrima* TK1 showed stronger cytotoxicity and genotoxicity against both tested cell lines than metabolites of the *L. plantarum* KB2 strain. More work is needed to understand how the presence of biocontrol agents in the environment can impact biodiversity. It is important not only to examine the effects of individual natural factors but also to explore their interactions with other biocontrol agents. Of course, any analytical approach must take into account the natural high variability of samples, such as plant extracts. Our research focused on Polish plants and their extracts. Any further studies must therefore take into account the geographical and climatic variability of the raw material used. In addition, future studies should aim to expand the use of in vitro cell line models while incorporating in vivo investigations, particularly in insect systems, to provide a more comprehensive understanding of these interactions and their implications. Our in vitro findings provide a foundation for further research. Future studies incorporating in vivo validation will be our next step.

## Figures and Tables

**Table 1:** Composition of volatile compounds in sage (*Salvia officinalis* L.) aqueous extract.

Component	KI *	Composition [%]
Hydroxyacetone	709	5.14
Eucalyptol	1016	17.74
α-Thujone	1084	24.24
β-Thujone	1096	4.36
Camphor	1118	31.00
Borneol	1148	2.59
2-Metoksy-4-vinylphenol	1283	10.78
Total unidentified compounds	-	4.13

* KI—Kovats index.

**Table 2:** Composition of volatile compounds in turmeric (*Curcuma longa* L.) aqueous extract.

Component	KI *	Composition [%]
Ethanol	-	13.92
Trimethylamine	-	1.19
1-Hydroxy-2-propanone	660	21.91
2,3-Butanediol	779	9.06
Cumene	911	2.05
Eucalyptol	1017	2.12
α-Curcumene	1469	1.23
β-Sesquiphellandrene	1514	1.91
Ar-Turmerone	1633	18.66
Tumerone	1643	2.71
Curlone	1674	7.98
Total unidentified compounds	-	17.25

* KI—Kovats index.

**Table 3:** Cytotoxic (IC_50_) and non-cytotoxic (IC_0_) values of plant aqueous extracts against insect Sf-9 and human HeLa cell lines.

Plant Extract	IC_0_ [mg/mL]		IC_50_ [mg/mL]	
	Sf-9	HeLa	Sf-9	HeLa
Garlic (*Allium sativum* L.)	<0.39	≤125	41.6	203 *
Turmeric (*Curcuma longa* L.)	<0.39	≤62.5	145	191
Sage (*Salvia officinalis* L.)	≤62.5	≤25	147	49.6
Clove (*Syzygium aromaticum* L. Merr. & Perry)	<0.78	≤0.78	216	53.3
λ-Cyhalothrin	<0.0004	<0.002	0.2	2.0

* IC_20._

**Table 4:** Cytotoxic (IC_50_) and non-cytotoxic (IC_0_) values for *L. plantarum* KB2 LAB 03 and *M. pulcherrima* TK1 metabolites cultivated in different media against insect Sf-9 and human HeLa cell lines.

Microbial Metabolites	IC_0_ [mg/mL]		IC_50_ [mg/mL]	
	Sf-9	HeLa	Sf-9	HeLa
*L. plantarum*, MRS, pH 4.0	<0.001	<10	40.6	73.9
*L. plantarum*, MRS, pH 7.0	<0.001	<0.1	110.3	75.5
*L. plantarum*, AM, pH 4.0	<0.001	<0.1	34.2	99.2
*L. plantarum*, AM, pH 7.0	<0.001	≤0.1	167.7	96.7
*M. pulcherrima*, YPG, pH 5.9	<0.001	<0.1	2.2	42.9
*M. pulcherrima*, YPG, pH 7.0	<0.001	<0.01	5.5	30.3
*M. pulcherrima*, sYP, pH 6.2	≤200	≤100	nd *	nd
*M. pulcherrima*, sYP, pH 7.0	<100	≤200	180.4	nd
*M. pulcherrima*, sYPG, pH 5.4	<0.01	<0.001	0.8	1.9
*M. pulcherrima*, sYPG, pH 7.0	<0.1	<0.1	0.1	0.8

* nd—not detected.

**Table 5:** Genotoxicity of plant aqueous extracts against the insect Sf-9 cell line. Number sign (^#^) denotes values that are statistically significant relative to the negative control. The same letters denote statistically significant differences between results at the same tested concentration (ANOVA, *p* ≤ 0.05).

Extract	Extract Concentration [mg/mL]		
	31.3	62.5	125
	% DNA in the Comet Tail (±SEM)		
Garlic (*Allium sativum* L.)	51.63 ± 3.78 ^#^	48.01 ± 3.98 ^#,a,b,c^	nt *
Turmeric (*Curcuma longa* L.)	nt	32.60 ± 3.02 ^#,a,d^	39.09 ± 3.49 ^#,f,g^
Sage (*Salvia officinalis* L.)	nt	30.60 ± 2.05 ^#,b,e^	27.49 ± 4.11 ^#,f^
Clove (*Syzygium aromaticum* L. Merr. & Perry)	nt	20.17 ± 2.51 ^#,c,d,e^	22.84 ± 3.48 ^#,g^

* nt—not tested.

**Table 6:** Genotoxicity of plant aqueous extracts against model human HeLa cell line. Number sign (^#^) denotes values that are statistically significant relative to the negative control; the same letters denote statistically significant differences between results at the same tested concentration (ANOVA, *p* ≤ 0.05).

Extract	Extract Concentration [mg/mL]			
	25	50	62.5	250
	% DNA in the Comet Tail (±SEM)			
Garlic (*Allium sativum* L.)	nt *	nt	4.52 ± 1.26	10.51 ± 2.53 ^#^
Turmeric (*Curcuma longa* L.)	nt	nt	3.97 ± 0.51	6.85 ± 0.75
Sage (*Salvia officinalis* L.)	15.91 ± 4.00 ^#^	24.29 ± 3.11 ^#,a^	nt	nt
Clove (*Syzygium aromaticum* L. Merr. & Perry)	11.58 ± 1.61 ^#^	44.55 ± 4.78 ^#,a^	nt	nt

* nt—not tested.

**Table 7:** Genotoxicity of *L. plantarum* KB2 LAB 03 metabolites after cultivation in different media against the insect Sf-9 cell line. Number sign (^#^) denotes values that are statistically different from the negative control; the same letters denote statistically significant differences between results at the same tested concentrations (ANOVA, *p* ≤ 0.05).

Culture Medium	Metabolites Concentration [mg/mL]		
	10	50	100
	% DNA in the Comet Tail (±SEM)		
MRS, pH 4.0	39.56 ± 3.41 ^#,a^	42.05 ± 3.33 ^#,b^	nt *
MRS, pH 7.0	44.86 ± 4,26 ^#^	41.95 ± 3.57 ^#,c^	nt
AM, pH 4.0	49.90 ± 2.85 ^#,a^	54.52 ± 4.45 ^#,b,c^	nt
AM, pH 7.0	nt	43.78 ± 3.40 ^#^	45.67 ± 3.76 ^#^

* nt—not tested.

**Table 8:** Genotoxicity of *M. pulcherrima* TK1 metabolites after cultivation in different media against the insect Sf-9 cell line. Number sign (^#^) denotes values that are statistically different from the negative control; the same letters denote statistically significant differences between results at the same tested concentrations (ANOVA, *p* ≤ 0.05).

Culture Medium	Metabolites Concentration [mg/mL]				
	0.01	0.1	1.0	50	100
	% DNA in the Comet Tail (±SEM)				
YPG, pH 5.9	nt *	51.02 ± 3.66 ^#,a^	49.51 ± 4.06 ^#,b^	nt	nt
YPG, pH 7.0	nt	38.59 ± 4.12 ^#,a^	38.83 ± 4.18 ^#,c^	nt	nt
sYP, pH 6.2	nt	44.58 ± 3.38 ^#^	61.21 ± 3.53 ^#,b,c^	nt	nt
sYP, pH 7.0	nt	nt	nt	51.91 ± 4.02 ^#^	57.97 ± 4.17 ^#^
sYPG, pH 5.4	45.31 ± 3.63 ^#^	45.54 ± 3.77 ^#^	nt	nt	nt
sYPG, pH 7.0	46.16 ± 4.06 ^#^	49.78 ± 3.96 ^#^	nt	nt	nt

* nt—not tested.

**Table 9:** Genotoxicity of *L. plantarum* KB2 LAB 03 metabolites after cultivation in different media against the human HeLa cell line. Number sign (^#^) denotes significant differences relative to the negative control.

Culture Medium	Metabolite Concentration [mg/mL]	
	50	100
	% DNA in the Comet Tail (±SEM)	
MRS, pH 4.0	5.26 ± 2.20	12.09 ± 3.36 ^#^
MRS, pH 7.0	5.03 ± 1.86	12.53 ± 3.87 ^#^
AM, pH 4.0	6.61 ± 2.22	10.32 ± 3.36 ^#^
AM, pH 7.0	8.53 ± 2.30 ^#^	7.98 ± 2.30 ^#^

**Table 10:** Genotoxicity of *M. pulcherrima* TK1 metabolites after cultivation in different media against the human HeLa cell line. Number sign (^#^) denotes values that are statistically significant relative to the negative control.

Culture Medium	Metabolite Concentration [mg/mL]					
	0.1	1.0	10	50	100	200
	% DNA in the Comet Tail (±SEM)					
YPG, pH 5.9	nt *	nt	nt	5.56 ± 2.04	7.52 ± 2.81	nt
YPG, pH 7.0	nt	6.37 ± 1.80	7.88 ± 2.44 ^#^	nt	nt	nt
sYP, pH 6.2	nt	nt	nt	nt	6.35 ± 2.46	14.99 ± 4.27 ^#^
sYP, pH 7.0	nt	nt	nt	nt	5.70 ± 2.09	6.35 ± 2.27
sYPG, pH 5.4	nt	7.66 ± 3.10 ^#^	7.09 ± 2.48 ^#^	nt	nt	nt
sYPG, pH 7.0	5.43 ± 2.13	10.77 ± 3.82 ^#^	nt	nt	nt	nt

* nt—not tested.

## Data Availability

The data presented in this study are available in this article and are available from the corresponding author upon reasonable request.

## References

[1] Ayaz M., Li C.H., Ali Q., Zhao W., Chi Y.K., Shafiq M., Ali F., Yu X.Y., Yu Q., Zhao J.T. (2023). Bacterial and fungal biocontrol agents for plant disease protection: Journey from lab to field, current status, challenges, and global perspectives. Molecules.

[2] Deepa N., Achar P.N., Sreenivasa M.Y. (2021). Current perspectives of biocontrol agents for management of *Fusarium verticillioides* and its fumonisin in cereals—A review. J. Fungi.

[3] Perczak A., Goliński P., Bryła M., Waśkiewicz A. (2018). The efficiency of lactic acid bacteria against pathogenic fungi and mycotoxins. Arh Hig Rada Toksikol..

[4] Sadiq F.A., Yan B., Tian F., Zhao J., Zhang H., Chen W. (2019). Lactic acid bacteria as antifungal and anti-mycotoxigenic agents: A comprehensive review. Compr. Rev. Food Sci. Food Saf..

[5] Steglińska A., Kołtuniak A., Motyl I., Berłowska J., Czyżowska A., Cieciura-Włoch W., Okrasa M., Kręgiel D., Gutarowska B. (2022). Lactic acid bacteria as biocontrol agents against potato (*Solanum tuberosum* L.) pathogens. Appl. Sci..

[6] Sipiczki M. (2020). *Metschnikowia pulcherrima* and related pulcherrimin-producing yeasts: Fuzzy species boundaries and complex antimicrobial antagonism. Microorganisms.

[7] Pawlikowska E., James S.A., Breierova E., Antolak H., Kregiel D. (2019). Biocontrol capability of local *Metschnikowia* sp. isolates. Antonie Van Leeuwenhoek.

[8] Steglińska A., Kołtuniak A., Berłowska J., Czyżowska A., Szulc J., Cieciura-Włoch W., Okrasa M., Kręgiel D., Gutarowska B. (2022). *Metschnikowia pulcherrima* as a biocontrol agent against potato (*Solanum tuberosum*) pathogens. Agronomy.

[9] Šernaitė L. (2017). Plant extracts: Antimicrobial and antifungal activity and appliance in plant protection (Review). Sodinink. Daržinink..

[10] Steglińska A., Bekhter A., Wawrzyniak P., Kunicka-Styczyńska A., Jastrząbek K., Fidler M., Śmigielski K., Gutarowska B. (2022). Antimicrobial activities of plant extracts against *Solanum tuberosum* L. phytopathogens. Molecules.

[11] Steglińska A., Sulyok M., Janas R., Grzesik M., Liszkowska W., Kręgiel D., Gutarowska B. (2023). Metabolite formation by fungal pathogens of potatoes (*Solanum tuberosum* L.) in the presence of bioprotective agents. Int. J. Environ. Res. Public Health.

[12] Amichot M., Bertrand C., Chauvel B., Corio-Costet M.F., Martin-Laurent F., Le Perchec S., Mamy L. (2024). Natural products for biocontrol: Review of their fate in the environment and impacts on biodiversity. Environ. Sci. Pollut Res. Int..

[13] He X., Lu L., Huang P., Yu B., Peng L., Zou L., Ren Y. (2023). Insect cell-based models: Cell line establishment and application in insecticide screening and toxicology research. Insects.

[14] Schymanski E.L., Gallampois C.M., Krauss M., Meringer M., Neumann S., Schulze T., Wolf S., Brack W. (2012). Consensus structure elucidation combining GC/EI-MS, structure generation, and calculated properties. Anal. Chem..

[15] Greff B., Sáhó A., Lakatos E., Varga L. (2023). Biocontrol activity of aromatic and medicinal plants and their bioactive components against soil-borne pathogens. Plants.

[16] López-Cabeza R., Rodríguez-Sabina S., Reyes C.P., Expósito D.G., Giménez C., Jiménez I.A., Cabrera R., Bazzocchi I.L. (2024). Bio-guided isolation of aromatic abietane diterpenoids from *Salvia canariensis* as biopesticides in the control of phytopathogenic fungi. Pest Manag. Sci..

[17] Vaughn C.J. (2012). Drugs and lactation database: LactMed. J. Electron. Resour. Med. Libr..

[18] Ivanov M., Kannan A., Stojković D.S., Glamočlija J., Calhelha R.C., Ferreira I.C.F.R., Sanglard D., Soković M. (2021). Camphor and eucalyptol-anticandidal spectrum, antivirulence effect, efflux pumps interference and cytotoxicity. Int. J. Mol. Sci..

[19] Fotiadou E., Panou E., Graikou K., Sakellarakis F.N., Chinou I. (2023). Volatiles of all native *Juniperus* species growing in Greece-antimicrobial properties. Foods.

[20] Morkeliūnė A., Rasiukevičiūtė N., Šernaitė L., Valiuškaitė A. (2021). The use of essential oils from thyme, sage and peppermint against *Colletotrichum acutatum*. Plants.

[21] Sigma Aldrich Data Sheet 2-Metoksy-4-vinylphenol. https://www.sigmaaldrich.com.

[22] Senouci H., Benyelles N.G., Dib M.E.A., Costa J., Muselli A. (2020). Chemical composition and combinatory antifungal activities of *Ammoides verticillata*, *Allium sativum* and *Curcuma longa* essential oils against four fungi responsible for tomato diseases. Comb. Chem. High Throughput Screen..

[23] Snetkov P., Rogacheva E., Kremleva A., Morozkina S., Uspenskaya M., Kraeva L. (2022). In-vitro antibacterial activity of curcumin-loaded nanofibers based on hyaluronic acid against multidrug-resistant ESKAPE pathogens. Pharmaceutics.

[24] Kampf G. (2018). Efficacy of ethanol against viruses in hand disinfection. J. Hosp. Infect..

[25] Genersch E., Gisder S., Hedtke K., Hunter W.B., Möckel N., Müller U. (2013). Standard methods for cell cultures in *Apis mellifera* research. J. Apic. Res..

[26] Islam M.S., Kusumoto Y., Al-Mamun M.A. (2011). Cytotoxicity and cancer (HeLa) cell killing efficacy of aqueous garlic (*Allium sativum*) extract. J. Sci. Res..

[27] Bagul M., Kakumanu S., Wilson T.A. (2015). Crude garlic extract inhibits cell proliferation and induces cell cycle arrest and apoptosis of cancer cells in vitro. J. Med. Food.

[28] Jasamai M., Hui C.S., Azmi N., Kumolosasi E. (2016). Effect of *Allium sativum* (garlic) methanol extract on viability and apoptosis of human leukemic cell lines. Trop. J. Pharm. Res..

[29] Szychowski K.A., Binduga U.E., Rybczyńska-Tkaczyk K., Leja M.L., Gmiński J. (2018). Cytotoxic effects of two extracts from garlic (*Allium sativum* L.) cultivars on the human squamous carcinoma cell line SCC-15. Saudi J. Biol. Sci..

[30] Szychowski K.A., Rybczyńska-Tkaczyk K., Gaweł-Bęben K., Swieca M., Karas M., Jakuczyk A., Matysiak M., Binduga U.E., Gminski J. (2018). Characterization of active compounds of different garlic (*Allium sativum* L.) cultivars. Pol. J. Food Nutr. Sci..

[31] Babu B.C., Sunil A., Mukunda A., Pynadath M.K., Aswathy A.M. (2020). Anti-cancer potency of garlic (*Allium sativum*) extract in comparison to 5-fluorouracil—An in vitro study. Oral Maxillofac. Patho. J..

[32] Țigu A.B., Moldovan C.S., Toma V.-A., Farcaș A.D., Moț A.C., Jurj A., Fischer-Fodor E., Mircea C., Pârvu M. (2021). Phytochemical analysis and in vitro effects of *Allium fistulosum* L. and *Allium sativum* L. Extracts on human normal and tumor cell lines: A comparative study. Molecules.

[33] Yan T., Zhao Y., Jiang Z., Chen J. (2022). Acetaldehyde induces cytotoxicity via triggering mitochondrial dysfunction and overactive mitophagy. Mol. Neurobiol..

[34] Yan T., Zhao Y. (2020). Acetaldehyde induces phosphorylation of dynamin-related protein 1 and mitochondrial dysfunction via elevating intracellular ROS and Ca(2+) levels. Redox Biol..

[35] Hussain A., Sasidharan S., Ahmed T., Ahmed M., Sharma C. (2009). Clove (*Syzygium aromaticum*) extract potentiates gemcitabine cytotoxic effect on human cervical cancer cell line. Int. J. Cancer Res..

[36] Hussein N.H., Maeah R.K., Sharba Z.A., Aasoon B., Sulaiman G.M., Ali A.A., Taha A., Jwad K.H. (2019). Cytotoxic, antioxidant and antibacterial activities of crude extract of *Syzygium aromaticum* plant. Plant Arch..

[37] Yassin M.T., Al-Askar A.A., Abdel-Fattah Mostafa A., El-Sheikh M.A. (2020). Bioactivity *of Syzygium aromaticum* (L.) Merr. & L.M. Perry extracts as potential antimicrobial and anticancer agents. J. King Saud Univ.-Sci..

[38] Imade R.O., Ayinde B.A. (2021). GC-MS analysis and in vitro cytotoxic activity of *Syzygium aromaticum* volatile oil and its major constituent–eugenol. J. Sci. Pract. Pharm..

[39] Nasomyon T., Charoonratana T. (2021). Comparative chemical composition and *in-vitro* cytotoxic activity of *Syzygium aromaticum* against selected cancer cell lines. Trop. J. Nat. Prod. Res..

[40] Mekky A.E., Emam A.E., Selim M.N., Abdelmouty E.S., Khedr M. (2024). Antibacterial and antineoplastic MCF-7 and Hep-G2 characteristics of the methanolic (80%) clove (*Syzygium aromaticum* L.) extract. Biomass Conv. Bioref..

[41] Pasri P., Mermillod P., Khempaka S. (2023). Antioxidant properties and cytotoxic effects of selected edible plants in Southeast Asia for further use as phytogenic antioxidant additives. Saudi J. Biol. Sci..

[42] Das A., K H., Kannan SK D., Raj K H., Jayaprakash B. (2018). Evaluation of therapeutic potential of eugenol-a natural derivative of *Syzygium aromaticum* on cervical cancer. Asian Pac. J. Cancer Prev..

[43] Abdulrahman M.D., Hama H.A. (2023). Anticancer of genus *Syzygium*: A systematic review. Explor. Target Antitumor Ther..

[44] Kunwar A., Barik A., Mishra B., Rathinasamy K., Pandey R., Priyadarsini K.I. (2008). Quantitative cellular uptake, localization and cytotoxicity of curcumin in normal and tumor cells. Biochim. Biophys. Acta.

[45] Yue G.G., Cheng S.W., Yu H., Xu Z.S., Lee J.K., Hon P.M., Lee M.Y., Kennelly E.J., Deng G., Yeung S.K. (2012). The role of turmerones on curcumin transportation and P-glycoprotein activities in intestinal Caco-2 cells. J. Med. Food.

[46] Ismail N.E., Abdul Samad M., Bustami Effendi T.J., Hazizul Hasan M. Cytotoxicity studies of aqueous extracts of *Curcuma longa* and *Quercus infectoria*. Proceedings of the International Conference on Science and Social Research 2010.

[47] Lawal R., James A.B., Shaibu R., Okoye O.P., Odetunde S.A., Ajibare A.C. (2022). Cytotoxic effects of *Curcuma longa* leaves on MCF-7 and HepG2 cells. Fountain J. Natural Appl. Sci..

[48] Abualhasan M., Jaradat N., Hawash M., Shraim N., Asaad M., Mousa A., Mousa Z., Tobeh R., Mlitat B. (2023). Chromatographic analysis of the chemical composition and anticancer activities of *Curcuma longa* extract cultivated in Palestine. Open Life Sci..

[49] Firoz H.M., Nanjundaiah S., Sadashiva C.T., Neethumol B., Rashmi Y., Sreedrisya A.K. (2023). Antiproliferative activity and apoptosis-inducing mechanism of *Curcuma longa* (Turmimax^®^) on HeLa cell lines. Braz. J. Biol..

[50] Ji M., Choi J., Lee J., Lee Y. (2004). Induction of apoptosis by Ar-turmerone on various cell lines. Int. J. Mol. Med..

[51] Sudarshan K., Yarlagadda S., Sengupta S. (2024). Recent Advances in the Synthesis of Diarylheptanoids. Chem. Asian J..

[52] Sudarshan K., Perumal G., Aidhen I.S., Doble M. (2018). Synthesis of Unsymmetrical Linear Diarylheptanoids and their Enantiomers and Antiproliferative Activity Studies. Eur. J. Org. Chem..

[53] Jiang Y., Zhang L., Rupasinghe H.P. (2017). Antiproliferative effects of extracts from *Salvia officinalis* L. and *Saliva miltiorrhiza* Bunge on hepatocellular carcinoma cells. Biomed. Pharmacother..

[54] Zare Shahneh F., Valiyari S., Baradaran B., Abdolalizadeh J., Bandehagh A., Azadmehr A., Hajiaghaee R. (2013). Inhibitory and cytotoxic activities of *Salvia officinalis* L. extract on human lymphoma and leukemia cells by induction of apoptosis. Adv. Pharm. Bull..

[55] Afonso A.F., Pereira O.R., Fernandes Â., Calhelha R.C., Silva A.M.S., Ferreira I.C.F.R., Cardoso S.M. (2019). Phytochemical composition and bioactive effects of *Salvia africana*, *Salvia officinalis* ‘Icterina’ and *Salvia mexicana* aqueous extracts. Molecules.

[56] Izham M.N.M., Hussin Y., Rahim N.F.C., Aziz M.N.M., Yeap S.K., Rahman H.S., Masarudin M.J., Mohamad N.E., Abdullah R., Alitheen N.B. (2021). Physicochemical characterization, cytotoxic effect and toxicity evaluation of nanostructured lipid carrier loaded with eucalyptol. BMC Complement. Med. Ther..

[57] Nikolić B., Vasilijević B., Mitić-Ćulafić D., Vuković-Gačić B., Knežević-Vukćević J. (2015). Comparative study of genotoxic, antigenotoxic and cytotoxic activities of monoterpenes camphor, eucalyptol and thujone in bacteria and mammalian cells. Chem. Biol. Interact..

[58] Lopes A.I.F., Monteiro M., Araújo A.R.L., Rodrigues A.R.O., Castanheira E.M.S., Pereira D.M., Olim P., Fortes A.G., Gonçalves M.S.T. (2020). Cytotoxic plant extracts towards insect cells: Bioactivity and nanoencapsulation studies for application as biopesticides. Molecules.

[59] Laborde M.R.R., Larramendy M.L., Soloneski S. (2023). Cytotoxic and genotoxic profiles of the pyrethroid insecticide lambda-cyhalothrin and its microformulation Karate^®^ in CHO-K1 cells. Mutat. Res. Genet. Toxicol. Environ. Mutagen.

[60] Xu X., Yu Y., Ling M., Ares I., Martínez M., Lopez-Torres B., Maximiliano J.E., Martínez-Larrañaga M.R., Wang X., Anadón A. (2023). Oxidative stress and mitochondrial damage in lambda-cyhalothrin toxicity: A comprehensive review of antioxidant mechanisms. Environ. Pollut..

[61] Pham C.H., Lee J.-E., Yu J., Lee S.H., Yu K.-R., Hong J., Cho N., Kim S., Kang D., Lee S. (2021). Anticancer effects of propionic acid inducing cell death in cervical cancer cells. Molecules.

[62] Nowak A., Zakłos-Szyda M., Rosicka-Kaczmarek J., Motyl I. (2022). Anticancer potential of post-fermentation media and cell extracts of probiotic strains: An in vitro study. Cancers.

[63] Haghshenas B., Abdullah N., Nami Y., Radiah D., Rosli R., Khosroushahi A.Y. (2014). Different effects of two newly-isolated probiotic *Lactobacillus plantarum* 15HN and *Lactococcus lactis* subsp. *lactis* 44Lac strains from traditional dairy products on cancer cell lines. Anaerobe.

[64] Orlando A., Refolo M.G., Messa C., Amati L., Lavermicocca P., Guerra V., Russo F. (2012). Antiproliferative and proapoptotic effects of viable or heat-killed *Lactobacillus paracasei* IMPC2.1 and *Lactobacillus rhamnosus* GG in HGC-27 gastric and DLD-1 colon cell lines. Nutr. Cancer.

[65] Nowak A., Paliwoda A., Błasiak J. (2019). Anti-proliferative, pro-apoptotic and anti-oxidative activity of *Lactobacillus* and *Bifidobacterium* strains: A review of mechanisms and therapeutic perspectives. Crit. Rev. Food Sci. Nutr..

[66] Garbacz K. (2022). Anticancer activity of lactic acid bacteria. Semin. Cancer. Biol..

[67] El-Beltagi H.S., El-Mahdy O.M., Mohamed H.I., El-Ansary A.E. (2022). Antioxidants, antimicrobial, and anticancer activities of purified chitinase of *Talaromyces funiculosus* strain CBS 129594 biosynthesized using crustacean bio-wastes. Agronomy.

[68] Upadhyay T.K., Trivedi R., Khan F., Al-Keridis L.A., Pandey P., Sharangi A.B., Alshammari N., Abdullah N.M., Yadav D.K., Saeed M. (2022). In vitro elucidation of antioxidant, antiproliferative, and apoptotic potential of yeast-derived β-1,3-glucan particles against cervical cancer cells. Front. Oncol..

[69] Abbasi A., Homayouni Rad A., Aghebati Maleki L., Samadi Kafil H., Baghbanzadeh A. (2023). Cytotoxic potentials of cell-free supernatant derived from *Lactobacillus casei* CRL431 on HCT-116 and HT-29 human colon cancer cell lines. Biointerface Res. Appl. Chem..

[70] Sekar N., Sundaramoorthy R., Majumdar S., Bhuyan K., Das J., Abilash V.G. (2015). Determination of allicin in *Allium sativum* using high performance liquid chromatography and study of genotoxic effect on human leukocytes. Asian J. Pharm. Clin. Res..

[71] Waqar Z., Sajjad U.R., Ahsan N., Safdar I., Qurratulain A., Majeed Q., Maqbool A., Waseem Abbas M. (2019). Genotoxicity assessment in aqueous and alcoholic extracts of *Allium sativum*, *Zingiber officinale* and *Trachyspermum ammi*. Curr. Trends Biomed. Eng. Biosci..

[72] Abraham K., Gürtler R., Berg K., Heinemeyer G., Lampen A., Appel K.E. (2011). Toxicology and risk assessment of 5-hydroxymethylfurfural in food. Mol. Nutr. Food Res..

[73] Severin I., Dumont C., Jondeau-Cabaton A., Graillot V., Chagnon M.C. (2010). Genotoxic activities of the food contaminant 5-hydroxymethylfurfural using different in vitro bioassays. Toxicol. Lett..

[74] Mohammadi Nejad S., Özgüneş H., Başaran N. (2017). Pharmacological and toxicological properties of eugenol. Turk. J. Pharm. Sci..

[75] Beltzig L., Frumkina A., Schwarzenbach C., Kaina B. (2021). Cytotoxic, genotoxic and senolytic potential of native and micellar curcumin. Nutrients.

[76] Cosquillo-Rafael M.F., Placencia-Medina M.D., Miranda-Tomasevich T.Y., Moreno-Hinojosa M., Retuerto-Figueroa M.G. (2020). In vitro cytotoxic and genotoxic effect of the crude and ethanolic extract from the rhizome of *Curcuma longa* L.. Rev. Peru Med. Exp. Salud Publica.

[77] Cao J., Jiang L.P., Liu Y., Yang G., Yao X.F., Zhong L.F. (2007). Curcumin-induced genotoxicity and antigenotoxicity in HepG2 cells. Toxicon.

[78] Zhao Y., Ye X., Xiong Z., Ihsan A., Ares I., Martínez M., Lopez-Torres B., Martínez-Larrañaga M.R., Anadón A., Wang X. (2023). Cancer metabolism: The role of ROS in DNA damage and induction of apoptosis in cancer cells. Metabolites.

[79] Blasiak J., Trzeciak A., Malecka-Panas E., Drzewoski J., Wojewódzka M. (2000). In vitro genotoxicity of ethanol and acetaldehyde in human lymphocytes and the gastrointestinal tract mucosa cells. Toxicol. In Vitro.

